# CowNflow: A dataset on nitrogen flows and balances in dairy cows fed maize forage or herbage-based diets

**DOI:** 10.1016/j.dib.2021.107393

**Published:** 2021-09-20

**Authors:** Manon Ferreira, Rémy Delagarde, Nadège Edouard

**Affiliations:** PEGASE, INRAE, Institut Agro, 16 Le Clos, Saint-Gilles 35590, France

**Keywords:** Dairy cow, Feeding, Nitrogen-use efficiency, Nitrogen-balance, Digestibility

## Abstract

Diet and animal characteristics have a significant impact on the nitrogen (N)-use efficiency of dairy cows. A dataset (CowNflow) was built that compiles 28 N-balance experiments with Holstein dairy cows from 1983 to 2019, corresponding to 414 individual N flows, for a wide range of diets and animal characteristics. The dataset is composed of six Microsoft® Excel files that correspond to six levels of information. The main file, “CowNflow_6_Cow_measurements” reports individual weekly measurements of dry matter intake, daily faeces and urine excretion, milk production and composition, cow characteristics, and chemical composition of diets, faeces, urine, and milk. These raw data were used to calculate the N-balance, N-use efficiency, and nutrients’ *in vivo* total-tract digestibility. The experiments, conducted under standardised conditions, had multiple aims and offered a wide range of diets. Consequently, each diet is classified according to the main forage offered, resulting in six diet types: (1) maize forage (maize silage or dehydrated maize) alone, (2) maize forage and dehydrated lucerne, (3) maize forage and grass hay, (4) maize forage and freshly cut herbage, (5) freshly cut herbage alone, and (6) dehydrated herbage. The other five Excel files provide supplementary information at larger scales and describe experiment characteristics, experimental treatments, offered feeds along with their chemical composition, ingredient composition of compound feeds, and cow characteristics. This dataset can be used to better understand animal and dietary determinants of N-use efficiency and the origin of N losses to the environment, to identify feeding strategies that reduce protein-rich concentrate use, and to decrease environmental impacts of dairy farming with a variety of foraging systems.

## Specifications Table

**Table:** 

Subject	Animal Science and Zoology
Specific subject area	Individual N-balance, N-use efficiency, and digestibility in relation to dairy cow and diet characteristics
Type of data	Table
How data were acquired	Data were acquired at the INRAE experimental dairy farm of Méjusseaume (Le Rheu, Brittany, France) from 1983 to 2019 with Holstein dairy cows. The data originate from 28 experiments conducted under standardised conditions, for 10 weeks on average, with 3–6 cows per experiment. Experiments were divided into 2–5 periods depending on the number of treatments studied; most experiments were Latin-square designs. Each cow × period data were thus characterised by a specific treatment (usually dietary). For each experiment, the dataset reports individual measurements of dry matter intake (weight of feeds offered and refused), daily faeces and urine excretion (total collected and weighed), milk production and composition, and chemical composition of feeds, faeces, urine, and milk, including mainly N concentrations. Data in the dataset were averaged over the 4, 5 measurement days (depending on the experiment) of each period.
Data format	Raw Analyzed
Parameters for data collection	The dataset contains only experiments for which daily N intake, N exported in milk, and faecal N excretion were measured. Thus, the data provide at least dry matter intake, crude protein concentration of each feed, diet composition, milk production, milk true protein concentration, faeces excretion, and faecal N concentrations.
Description of data collection	Raw and analyzed data were collected for 28 experiments directly from the INRAE researchers responsible for the experiments. Data were pooled in a dataset composed of six Microsoft® Excel files, each of which presents one kind of information: description of the experiments, treatments, feeds, compound feed ingredients, cow characteristics, and cow measurements.
Data source location	Experimental farm of PEGASE, INRAE, Institut Agro Méjusseaume, 35650 Le Rheu, Brittany France
Data accessibility	Repository name: Portail Data INRAE Data identification number: DOI Dataset CowNflow: 10.15454/FKDGTG Direct URL to data: 10.15454/FKDGTG

## Value of the Data

• Deep statistical analyses of previously acquired data are essential to provide new knowledge about determinants of the N-use efficiency of dairy cows. This large dataset pools measurements of complete N-balances at individual scale when the literature reports only averages at the treatment by experiment level. These *in vivo* measurements obtained in the same experimental conditions with the same measurement methods require specific equipment and skills.
• The data were obtained from dairy cows fed a variety of diet types, including a wide range of concentrate type and proportion, and a large range of diet N concentration. Theses diets also include contrasting forages, half of them being fresh herbage, for which data on urinary excretion are scarce in the literature.
• The data may be useful for researchers and nutritionists involved in dairy cow nutrition, digestion, energy balance, feed and N-use efficiency, and N excretion.
• The data provide extensive metadata on animals, diets’ ingredients and measurement methods that should help the interpretation of N and energy balance, feed and N-use efficiency. These data and metadata allow deep statistical analyses (e.g. meta-analyses) on balances and on their determinants. These analyses can help identify gaps in current knowledge and suggest new requirements for future studies.
• Better understanding of N flows in dairy cows fed a variety of diet types should improve estimates of N losses to the environment (air, water) depending on feeding systems or seasons, and thus help perform national inventories. It also should help to identify feeding strategies that reduce farm N inputs and increase the farm overall efficiency.
• These *in vivo* experimental data can finally be reused as an alternative or complementary approach and decrease future animal experimentation in the context of ethical use of animals (the Three Rs principles).

## 1 Data Description

The CowNflow dataset consists of six Microsoft® Excel files: “CowNflow_1_Experiments”, “CowNflow_2_Treatments”, “CowNflow_3_Feeds”, “CowNflow_4_Ingredients”, “CowNflow_5_Cows” and “CowNflow_6_Cow_measurements”. Each file includes a unique identifier (ID) per row that provides a link to the other files. Each ID concatenates multiple codes for each file: the experiment (starting with “*E*”), the treatment within the experiment (starting with “*T*”), the feed within the experiment (starting with “*F*”), the ingredient within the feed and experiment (starting with “*I*”), the cow within the experiment (starting with “*C*”), and the period within the experiment (starting with “*P*”). The codes end with an intra-experiment number, which provides each row with a unique ID.

The first file, “CowNflow_1_Experiments”, provides the following information, with one row per experiment (*N* = 28):

• Identification data, including the unique experiment ID per row (e.g. E02), the experiment number, and the year the experiment was performed
• The experiment's objectives
• The experimental design, with the number and length of periods, the number of treatments, and the number of cows
• Experimental conditions, with the number of diet distributions and number of milkings per day
• Sample-conservation conditions
• N-determination methods used per sample type
• References to scientific articles for published experiments

The second file, “CowNflow_2_Treatments”, contains one row per treatment within each experiment (N = 89) and provides:

• Identification data, with the unique treatment ID per row that concatenates the experiment and treatment codes within each experiment (e.g. E02_T1)
• Description of the treatment
• Data about animal feeding, with the feeding level as a percentage of voluntary intake

In this file, each treatment is associated with a “diet type” according to the main forage offered (proportions of dry matter for all forages offered in the diet, thus excluding concentrates). Six diet types were defined to describe all diets in this dataset (Tables 1 and 2):

• “Maize” for diets with ≥ 85% maize forage (maize silage or dehydrated maize)
• “Maize_Lucerne” for diets based on maize forage with ≥ 15% dehydrated lucerne
• “Maize_Hay” for diets based on maize forage with ≥ 15% hay
• “Maize_Fresh_herbage” for diets with mixed forage maize and freshly cut herbage (≥ 20% each)
• “Fresh_herbage” for diets with ≥ 85% freshly cut herbage
• “Dehydrated_herbage” for diets with ≥ 85% dehydrated herbage

**Table 1:** Criteria used to classify the diet types, expressed as the dry matter proportion of each forage category within the total forage offered in the diet, excluding concentrates.

	Diet type					
Forage category	Maize	Maize_ Lucerne	Maize_ Hay	Maize_Fresh_ herbage	Fresh_ herbage	Dehydrated_ herbage
Maize silage and dehydrated maize	≥0.85	≥0.50	≥0.50	≥0.20	<0.15	<0.15
Dehydrated lucerne	<0.15	≥0.15	<0.15	<0.15	<0.15	<0.15
Hay	<0.15	<0.15	≥0.15	<0.15	<0.15	<0.15
Fresh herbage	<0.15	<0.15	<0.15	≥0.20	≥0.85	<0.15
Dehydrated herbage	<0.15	<0.15	<0.15	<0.15	<0.15	≥0.85
Straw	<0.15	<0.15	<0.15	<0.15	<0.15	<0.15

**Table 2:** Main characteristics, feed proportions, and chemical composition of the diets for each diet type.

	Diet type					
	Maize	Maize_ Lucerne	Maize_ Hay	Maize_Fresh_ herbage	Fresh_ herbage	Dehydrated_ herbage
Number of experiments	10 (1)	4 (1)	1	1	11	1
Number of rows in the “CowNflow_6_Cow_measurements” file	163	56	22	24	234	25

Feed proportions in the diet, g/g DM						
Maize silage and dehydrated maize	0.728 ± 0.0728	0.433 ± 0.0847	0.495 ± 0	0.427 ± 0.1548	0	0
* min-max*	*0.482–0.828*	*0.370–0.671*	*0.495–0.495*	*0.213–0.643*	*0*	*0*
Dehydrated lucerne	0	0.234 ± 0.0399	0	0	0	0
* min-max*	*0*	*0.124–0.250*	*0*	*0*	*0*	*0*
Hay	0	0.011 ± 0.0182	0.145 ± 0	0	0	0.095 ± 0.1211
* min-max*	*0*	*0 – 0.040*	*0.145–0.145*	*0*	*0*	*0.089 – 0.119*
Fresh herbage	0	0	0	0.489 ± 0.1763	0.931 ± 0.1240	0
* min-max*	*0*	*0*	*0*	*0.245–0.735*	*0.577–1.000*	*0*
Dehydrated herbage	0	0	0	0	0	0.697 ± 0.0889
* min-max*	*0*	*0*	*0*	*0*	*0*	*0.651 – 0.872*
Straw	0	0.006 ± 0.0153	0	0	0.001 ± 0.0041	0
* min-max*	*0*	*0 – 0.050*	*0*	*0*	*0 – 0.019*	*0*
Concentrates	0.272 ± 0.0728	0.316 ± 0.0417	0.360 ± 0	0.084 ± 0.0217	0.068 ± 0.1212	0.208 ± 0.1011
* min-max*	*0.172 – 0.518*	*0.206 – 0.380*	*0.360 – 0.360*	*0.051 – 0.114*	*0 – 0.423*	*0.009 – 0.260*

Chemical composition of the diet						
DM, g/kg	368 ± 24.0	800 ± 117.3	893 ± 3.3	221 ± 45.3	182 ± 37.7	902 ± 3.3
* min-max*	*331 – 420*	*670 – 921*	*888 – 900*	*159 – 310*	*122 – 278*	*897 – 906*
OM, g/kg DM	938 ± 10.0	932 ± 4.7	934 ± 2.4	947 ± 10.3	892 ± 17.6	908 ± 7.1
* min-max*	*880 – 952*	*915 – 940*	*931 – 939*	*919 – 962*	*849 – 928*	*899 – 922*
N, g/kg DM	24.1 ± 3.88	24.9 ± 2.47	24.5 ± 3.87	25.1 ± 1.85	29.4 ± 8.02	26.8 ± 5.79
* min-max*	*17.2 – 38.4*	*18.7 – 29.0*	*18.3 – 30.0*	*22.8 – 29.4*	*16.6 – 47.5*	*22.2 – 38.6*
CP, g/kg DM	149 ± 22.6	155 ± 15.5	153 ± 24.2	157 ± 11.6	184 ± 50.0	167 ± 36.2
* min-max*	*107 – 217*	*117 – 181*	*114 – 187*	*142 – 184*	*103 – 296*	*138 – 241*
NDF, g/kg DM	386 ± 53.2	370 ± 30.1	NA	461 ± 23.0	448 ± 66.2	486 ± 45.6
* min-max*	*321 – 516*	*326 – 475*	*NA*	*413 – 496*	*271 – 560*	*422 – 540*
ADF, g/kg DM	184 ± 18.7	200 ± 27.5	NA	224 ± 9.7	229 ± 40.1	245 ± 35.8
* min-max*	*151 – 240*	*175 – 289*	*NA*	*207 – 240*	*142 – 303*	*195 – 305*

(1) One experiment had a treatment corresponding to the “Maize” diet type and another treatment that corresponded to the “Maize_Lucerne” diet type. For each variable, line 1: mean and standard deviation, line 2: min and max. DM = dry matter; OM = organic matter; N = nitrogen; CP = crude protein; NDF = neutral detergent fibre; ADF = acid detergent fibre.

The third file, “CowNflow_3_Feeds”, describes all of the feeds offered, including their chemical composition, with one row per feed within each experiment (*N* = 241). This file provides identification data with the unique feed ID per row, which concatenates the experiment and feed codes in the experiment (e.g. E02_F04). Each feed has a detailed name and is associated with either a forage or a concentrate category. Dehydrated and pelleted forages are considered forages in this database.

The seven forage categories are maize silage, dehydrated maize, fresh herbage, hay, dehydrated herbage, dehydrated lucerne, and straw. The concentrate categories in this dataset correspond to six concentrate categories of INRA (2018) feed tables [1], cereals, oil seed meals, compound feeds, other plant products, minerals and vitamins, and other products.

This file provides the chemical composition of all feeds, with their dry matter (DM), organic matter (OM), ash, crude protein (CP), and fibre (NDF, ADF, ADL) concentrations for nearly all feeds. Crude fibre and starch concentrations are measured less frequently.

The fourth file, “CowNflow_4_Ingredients”, provides the ingredient composition of compound feeds, with one row per ingredient within the compound feed (*N* = 387), and is composed of:

• Identification data with the unique ingredient ID per row, which concatenates the experiment and feed codes within the experiment and the ingredient code within the compound feed (e.g. E02_F04_I03)
• The ingredient's name
• The ingredient's category (corresponding to nine categories of INRA (2018) feed tables [1]): Cereals, cereal by-products, legume and oil seeds, oil seed meals, dehydrated lucerne, other plant products, oils and fat, minerals and vitamins, and other products.
• Its proportion in the corresponding compound feed (based on fresh matter)

The fifth file, “CowNflow_5_Cows”, provides cow characteristics, with one row per cow and per experiment (N = 137), and is composed of:

• Identification data with the unique cow ID per row, which concatenates the experiment and cow codes within experiment (e.g. E02_C5) and the cow number
• Key dates (birth, first calving, calving before and recalving after the experiment, drying off)
• The lactation number
• Cow characteristics (peak milk production, age at first calving, age at calving)

The sixth file, “CowNflow_6_Cow_measurements”, provides data per cow and per period within the experiment (*N* = 414) (Table 3). This file is divided into nine sections in columns. The section number is given at the beginning of the column name.

**Table 3:** Descriptive statistics of cow characteristics, nitrogen (N) intake, digestibility, and excretion in the “CowNflow_6_Cow_measurements” file of the CowNflow dataset.

Variable	n	Mean	Standard deviation	Median	Minimum	Maximum
Lactation number	414	3.2	1.70	3.0	1	8
Age (month)	414	64.0	23.71	56.3	30.9	132.2
Body weight (kg)	414	636	77.6	635	430	907
Lactation week	414	32.7	14.22	30.0	7.86	88.0
DM intake (kg/day)	414	18.1	3.89	18.5	8.02	29.6
Diet N concentration (g/kg DM)	414	26.1	5.89	24.7	16.6	47.5
N intake (g/day)	414	463	110.0	472	194	864
DM digestibility (g/kg)	414	720	46.4	714	611	857
N digestibility (g/kg)	414	702	68.5	700	506	856
Milk production (kg/day)	402	23.8	8.15	24.1	5.52	47.0
Milk N excretion (g/day)	403	124	36.8	126	35.8	218
Milk urea concentration (mg/dL)	180	21.5	10.06	19.5	2.49	48.1
Faecal DM excretion (kg/day)	414	5.17	1.706	5.43	1.46	9.45
Faecal N concentration (g/kg DM)	414	27.2	4.72	26.5	18.8	43.2
Faecal N excretion (g/day)	414	137	42.7	137	43.3	263
Urine excretion (kg/day)	390	23.4	11.83	20.2	6.53	69.5
Urinary N concentration (g/kg)	379	8.22	3.47	7.88	1.78	21.9
Urinary N excretion (g/day)	379	169	71.7	157	47.0	420
Urinary urea concentration (g/kg)	414	11.1	6.99	9.88	0.444	37.3
Basal plasma urea concentration (mg/dL)	158	20.8	14.73	16.4	1.95	53.7
N-balance (g/day)	414	40.4	34.29	36.3	-58.4	168
N-use efficiency (g/g)	414	0.270	0.0721	0.277	0.0851	0.442

DM = dry matter; N = nitrogen, n = number of rows.

Section 1 identifies each row with the unique cow measurement ID, which concatenates the experiment code and the cow and period codes within the experiment (e.g. E02_C5_P1). This section also provides the experiment ID, cow ID, and treatment ID, thus providing a link to the other files.

Section 2 provides cow characteristics during the experimental period, such as age, body weight (one weighing per period for 22 experiments or once per experiment for 6 experiments), physiological status (lactating or dry), lactation week, gestation status (pregnant or not), and gestation week. This dataset is composed essentially of data from lactating cows (403 rows).

Section 3 describes diet characteristics, with feed ID provided from the file “CowNflow_3_Feeds”, linked to the amount of each feed ingested. This section also provides proportions of the forages in the diet.

Section 4 describes the chemical composition of the ingested diet, with diet concentration and intake for DM, OM, ash, N, CP, NDF, ADF, and ADL. This section also provides the amount of water drunk daily and total water intake, when measured.

Section 5 contains daily faeces excretion and its composition, with faecal DM, OM, C, ash, N, CP, NDF, and ADF concentrations and excreted amounts. This section ends with *in vivo* total-tract digestibility of these nutrients and the diet concentrations of non-digestible N, CP, and OM (g/kg of DM intake).

Section 6 provides daily milk production, milk fat and true protein concentrations, N exported in milk, and milk urea concentration, when measured.

Section 7 provides daily urine excretion, its N concentration, and daily urinary N excretion. It also provides urinary urea concentration and excretion for some experiments.

Section 8 provides plasma urea concentration before the first meal distribution of the day (basal level) and 3, 4, or 6 h after that meal, when measured.

Section 9 provides calculated data related to N-balance and N-use efficiency.

## 2 Experimental Design, Materials and Methods

### 2.1 Materials and methods of the experiments

All experiments were conducted at the INRAE PEGASE experimental dairy farm (Le Rheu, Brittany, France), with similar experimental conditions and measurement methods. Cows were housed in tie stalls for the entire duration of the experiments, in controlled and mechanically ventilated rooms. They could see, smell, and hear each other. Cows were fed in individual troughs, had free access to water, and were milked twice a day directly in the room.

#### 2.1.1 Cows, treatments, experimental design, and conditions

Experiments were conducted with primiparous or multiparous Holstein cows at different lactation and gestation stages. Before each experiment, cows were fed the same diet and were characterised by their voluntary intake, body weight, and milk production and composition.

The main factors investigated in the experiments were:

• Concentrate amount, composition, or proportion in the diet
• Forage composition and proportion in the diet
• Diet N concentration
• Feed N degradability
• Frequency of feed distribution
• Feeding level (*ad libitum* vs. restricted feeding)

Most experiments were conducted according to Latin square or switchback designs, with each cow receiving one treatment per period and changing from one treatment to another between periods. Each period consisted of an adaptation phase of at least 5 days (10 days, on average) and a measurement phase. Along with intake measurements, all urine and faeces were collected for 4, 5 consecutive days in the measurement phase to estimate diet digestibility and daily N excretion and balance. All data provided in this dataset are averages of the measurement phases.

#### 2.1.2 Feed characteristics and intake

Offered feeds and refused diets were weighed daily and dried to determine their DM concentration, in order to calculate individual DM intake. Offered wet feeds (fresh herbage, silage) were dried at least once daily. Dry feeds (concentrates, dehydrated forage) were dried at least once weekly. Refused diets were dried once daily. The chemical compositions of feeds and refusals were determined to calculate the amount (g/day) of each nutrient offered, refused and, by their difference, eaten, in the diet (1). The concentration of each nutrient in the diet was calculated as the ratio of nutrient intake to total DM intake.

(1) $$Nutrient\;intake=\sum_{n}^{1}\left(OF\times NUT_{OF}\right)-\left(RD\times NUT_{RD}\right)$$

with *n* the number of feeds in the diet, OF the amount of each feed offered (kg DM/day), NUT_OF_ the concentration of the nutrient in each feed (g/kg DM), RD the amount of diet refused (kg DM/d), and NUT_RD_ the concentration of the nutrient in the refused diet (g/kg DM).

Chemical analyses of offered feed were performed at least once per experiment for concentrates and usually once per period for forages. For refusals, chemical analyses were performed on pooled samples per cow and per period.

Individual and mechanical water meters were used to record the amount of water drunk daily at the individual level. Total water intake was calculated as drunk water plus water provided in the diet.

#### 2.1.3 Milk production and composition

Individual milk production was recorded each day at each milking. Milk true protein and fat concentrations were measured for morning and afternoon milkings of the same day, for an average of 3 days per measurement period. Milk true protein and fat concentrations were measured by mid-infrared spectrophotometry (Milkoscan, Foss Electric, Hillerød, Denmark).

#### 2.1.4 Urine and faeces excretion and composition, and digestibility calculations

Cows were equipped with harnesses to collect all urine and faeces separately. Urine was immediately acidified with 500 ml of 20% H_2_SO_4_ to prevent ammonia volatilisation. Urine was weighed daily, and a sample of 0.5% or 1% of the total amount excreted was collected each day from each cow, depending on the year (0.5% from 1983 to 2012, 1% thereafter). Faeces were collected in a gutter behind the cow and weighed daily. Samples of 1–3% of the total fresh amount excreted were taken each day for each cow, depending on the experiment.

The chemical composition of faeces and urine was determined from pooled samples per cow and per period taken from the homogenised daily samples.

#### 2.1.5 Chemical analyses

Dry matter concentration was determined at the Méjusseaume farm from 1983 to 2013 by oven-drying feeds for 48 h and faeces for 48–72 h at 80 °C. For subsequent experiments, feeds and faeces were dried in a ventilated oven at 60 °C for 48 h and 72 h, respectively.

Most other chemical analyses were conducted at INRAE PEGASE (35590 Saint-Gilles, Brittany, France). OM concentration was determined by ashing in a muffle furnace at 500 or 550 °C for 5 or 6 h, depending on the experiment [2].

Fibre concentrations of feeds and faeces, corresponding to NDF, ADF and ADL fractions, were measured using a Fibertec extraction unit (Tecaton, Denmark) for experiments conducted from 1983 to 1993, and using a Fibersac extraction unit (Ankon Technology, Fairport, NY, USA) for subsequent experiments [2], [3], [4].

Nitrogen concentrations of feeds, faeces, milk, and urine were determined using the Kjeldahl method for the 18 experiments conducted from 1983 to 2002, and using the Dumas method on Leco equipment (Leco, St. Joseph, MI, USA) for the subsequent 10 experiments [2].

These chemical analyses were performed on dried or on frozen and lyophilised samples for feeds and refusals. They were performed on dried, frozen, or frozen and lyophilised samples for faeces, depending on the experiment. In addition to true protein, N concentrations were determined for fresh milk and fresh or frozen urine, depending on the experiment.

Milk, urine, and plasma urea concentrations were determined for 13, 13, and 9 experiments, respectively. Urea was measured from a colorimetric reaction assessed by a multi-parameter analyzer (AutoAnalyzer, Technicon Corporation, for experiments before 2002, then KONE Instruments 200 Corporation, Espoo, Finland, for subsequent experiments).

### 2.2 Materials and methods for building the dataset

#### 2.2.1 Data recovery and screening

Data related to protocols, experimental conditions, and measurements were collected directly from the INRAE researchers responsible for the experiments. The dataset was built from experiments that had at least the following data available: daily N intake, N exported in milk and faecal N excretion.

Some cow × period data resulting from outlier measurements (heifers or cows that were sick or drying up during the period) (*N* = 11), were not included in the dataset.

#### 2.2.2 Data calculation

The whole-tract digestibilities of DM, OM, N, NDF, and ADF (g/kg) were calculated from the amount of each component excreted in faeces (g/day) and ingested (g/day) (2):

(2) Digestibility=(intake−faecalexcretion)/intake×1000

Concentrations of non-digestible N, CP, and OM (all g/kg DM) in the diet were calculated as the ratio of faecal N, CP, or OM excretion (g/day) to the total intake (kg DM/day), respectively.

Feed and faecal CP concentrations were calculated by multiplying feed and faecal N concentrations, respectively, by 6.25.

In the database, N exported in milk was determined directly (12 experiments), estimated from milk true protein concentration (10 experiments), or estimated from milk true protein and urea concentrations (6 experiments).

N exported in milk (N milk, g/day) was estimated using milk true protein concentration (MPc, g/kg), milk production (MY, kg/day), and milk urea concentration (UREAc, mg/dL) using equations (3) or (4), depending on the data available [5]:

(3) Nmilk=MY×MPc/6.38+MY/1.032×UREAc/100×28/60+MY×0.125

(4) Nmilk=MY×(MPc+1.6)/6.38

These equations assume that milk true protein contains 15.7% N (1/6.38), milk specific-gravity is 1.032 kg/L [6], urea contains 46.7% N (28/60), milk contains 0.125 g per kg non-protein non-urea-N [7], and milk CP concentration equals milk true protein concentration plus 1.6 g/kg [8].

Nitrogen-use efficiency was calculated by dividing N exported in milk (g/day) by N intake (g/day). Nitrogen-balance was calculated from N intake (g/day), N exported in milk (g/day), urinary N excretion (N urine, g/day), and faecal N excretion (N faeces, g/day) (5):

(5) Nbalance=Nintake−Nmilk−Nurine−Nfaeces

Consequently, the N balance gathers both a part retained by the cow (for growth, maintenance and gestation if relevant), and an unaccounted part link to measurement errors and other sources of N excretion not considered.

#### 2.2.3 Data verification

All units and absolute values of the data provided in the CowNflow dataset were verified carefully using many graphs that combined all variables. We also checked that the N balance and N excretion values presented in the CowNflow dataset are in the range of published papers [9].

## 3 Interests and Potential Data Reuses

The CowNflow dataset compiles *in vivo* data, with N excretion measurements, when most of studies in the literatures generally estimate N excretion, due to the complexity of total faeces and urine collection. The originality of this dataset is to gather numerous N flow measurements on contrasting diets, including a large range of N content on fresh herbage or maize-based diets, all obtained in standardised conditions within a single research station. Theses N flow measurements at the individual scale may be useful for researchers in many fields to deeply analyze the influence of many parameters from the animals, the forage and the diets, considering the individual variability. More specifically, this dataset may improve the prediction equations on N excretion and partitioning and help identify feeding strategies to reduce N losses to the environment.

## Ethics Statement

Experiments were conducted in accordance with the French and European animal experimentation and animal welfare legislation applicable at the time that they were performed. Experiments E26, E27 and E28 were approved by the animal ethics committee (approval numbers: APAFIS#8548-2016120622288477-v3 for E26 and E27 experiments, APAFiS#16174-2018071817132587-v2 for E28 experiment). All experiments were conducted according to the “Three Rs” principles (Replacement, Reduction and Refinement) for the use of animals in research.

## Data Availability

CowNflow: A dataset on nitrogen flows and balances in dairy cows fed maize forage or herbage-based diets (Original data) (DataINRAE). https://doi.org/10.15454/FKDGTG.

## Funding

This work was performed within a Ph.D. thesis (Manon Ferreira) supported by the French National Research Institute for Agriculture, Food and Environment (INRAE – PHASE division) and the Brittany Region. The dataset was built as part of the EMIGRAZE project, supported by the French Environment and Energy Management Agency (ADEME).

## CRediT authorship contribution statement

**Manon Ferreira:** Conceptualization, Data curation, Validation, Writing – original draft. **Rémy Delagarde:** Conceptualization, Validation, Supervision, Writing – review & editing. **Nadège Edouard:** Conceptualization, Validation, Supervision, Writing – review & editing.

## Declaration of Competing Interest

The authors declare that they have no known competing financial interests or personal relationships which have, or could be perceived to have, influenced the work reported in this article.

## References

[1] Nozière P., Sauvant D., Delaby L. (2018).

[2] AOAC, (1990).

[3] Van Soest P.J. (1963). Use of detergents in the analysis of fibrous feeds. 2. A rapid method for the determination of fiber and lignin. J. Assoc. Off. Agric. Chem..

[4] Van Soest P.J., Robertson J.B., Lewis B.A. (1991). Methods for dietary fiber, neutral detergent fiber, and nonstarch polysaccharides in relation to animal nutrition. J. Dairy Sci..

[5] E. Cutullic, P. Faverdin, N. Edouard, J.L. Peyraud, Collaborative project FP7 RedNex, innovative and practical management approaches to reduce nitrogen excretion by ruminants. Merged Deliverables D7.2 (Report on the final validation of the shared data basis on N-balance) and D7.3 (Report of simulation to quantify the effect of the main factors affecting N-balance at cow level), 2013. https://cordis.europa.eu/project/id/211606/fr.

[6] Alais C. (1984).

[7] Walstra P., Geurts T.J., Noomen A., Jellema A., Van Boekel M.A.J.S. (1999).

[8] Faverdin P., Vérité R. (1998). Utilisation de la teneur en urée du lait comme indicateur de la nutrition protéique et des rejets azotés chez la vache laitière. Rencontres Recherches Ruminants.

[9] Castillo A.R., Kebreab E., Beever D.E., France J. (2020). A review of efficiency of nitrogen utilisation in lactating dairy cows and its relationship with environmental pollution. J. Anim. Feed Sci..

